# Significant Down-Regulation of “Biological Adhesion” Genes in Porcine Oocytes after IVM

**DOI:** 10.3390/ijms18122685

**Published:** 2017-12-11

**Authors:** Joanna Budna, Piotr Celichowski, Artur Bryja, Marta Dyszkiewicz-Konwińska, Michal Jeseta, Dorota Bukowska, Paweł Antosik, Klaus Peter Brüssow, Małgorzata Bruska, Michał Nowicki, Maciej Zabel, Bartosz Kempisty

**Affiliations:** 1 Department of Histology and Embryology, Poznan University of Medical Sciences, 60-781 Poznan, Poland; joanna.budna@wp.pl (J.B.); pcelichowski@ump.edu.pl (P.C.); mnowicki@ump.edu.pl (M.N.); mazab@ump.edu.pl (M.Z.); 2 Department of Anatomy, Poznan University of Medical Sciences, 60-781 Poznan, Poland; abryja@ump.edu.pl (A.B.); mdyszkiewicz@ump.edu.pl (M.D.-K.); bruska@ump.edu.pl (M.B.); 3 Department of Biomaterials and Experimental Dentistry, Poznan University of Medical Sciences, 60-812 Poznan, Poland; 4 Department of Obstetrics and Gynecology, University Hospital and Masaryk University, 602 00 Brno, Czech Republic; jeseta@gmail.com; 5 Veterinary Center, Nicolaus Copernicus University in Toruń, 87-100 Torun, Poland; lecznicapaw@gmail.com (D.B.); antosik67@wp.pl (P.A.); prof.bruessow@gmail.com (K.P.B.); 6 Department of Histology and Embryology, Wroclaw Medical University, 50-368 Wroclaw, Poland

**Keywords:** pig, oocytes, gamete biology, molecular biology, in vitro maturation (IVM)

## Abstract

Proper maturation of the mammalian oocyte is a compound processes determining successful monospermic fertilization, however the number of fully mature porcine oocytes is still unsatisfactory. Since oocytes’ maturation and fertilization involve cellular adhesion and membranous contact, the aim was to investigate cell adhesion ontology group in porcine oocytes. The oocytes were collected from ovaries of 45 pubertal crossbred Landrace gilts and subjected to two BCB tests. After the first test, only granulosa cell-free BCB^+^ oocytes were directly exposed to microarray assays and RT-qPCR (“before IVM” group), or first in vitro matured and then if classified as BCB^+^ passed to molecular analyses (“after IVM” group). As a result, we have discovered substantial down-regulation of genes involved in adhesion processes, such as: organization of actin cytoskeleton, migration, proliferation, differentiation, apoptosis, survival or angiogenesis in porcine oocytes after IVM, compared to oocytes analyzed before IVM. In conclusion, we found that biological adhesion may be recognized as the process involved in porcine oocytes’ successful IVM. Down-regulation of genes included in this ontology group in immature oocytes after IVM points to their unique function in oocyte’s achievement of fully mature stages. Thus, results indicated new molecular markers involved in porcine oocyte IVM, displaying essential roles in biological adhesion processes.

## 1. Introduction

During mammalian folliculogenesis and oogenesis the “ovarian follicle” differentiates from primordial, primary and secondary, into the fully organized Graafian follicle. These unique stages are accompanied by substantial growth and development of follicle [1], which is composed of differentiating theca cells (TCs) and granulosa cells (GCs). The TCs and GCs apart from forming follicular architecture, present endocrine activity, thus regulating proper function of follicular environment [2]. During each stage of oogenesis, the growing oocyte achieves the ability for development and maturation. The oocyte maturation is divided into two crucial steps: nuclear, which is associated with achievement of proper chromosomal configuration by the oocyte, and cytoplasmic, which is accompanied by storage of RNA and proteins for further embryo growth [3,4]. It was well recognized in several species of mammals that only properly structuralized mature oocyte (MII) may be successfully fertilized by spermatozoon, and each stage of early folliculogenesis and oogenesis influences further embryo growth and development [5,6,7]. Our previous experiments indicated an increased expression of genes involved in “Bone development”, “Cellular components of morphogenesis”, “BMP signaling pathway”, and “Cell migration”, which were intensively regulated during in vitro maturation of porcine oocytes (IVM) [8,9,10,11,12].

Cellular adhesion belongs to one of the most important biological features of cells growing in vivo and/or in vitro, since it is a crucial step in cell life or death, proper cell-to-cell communication, maintaining of membrane balance and induction of signalling/metabolic pathways [13]. Cellular adhesion is guaranteed by three main types of junctions. The first one, allowing formation of more complex structures involving cytoskeleton, are called adherens junctions (AJs). These include integrins, cadherins and desmosomes [14]. The second type, tight junctions, control ion and molecule flow through adjacent cells thanks to their selectivity [15,16]. Occludins and claudins are the most common proteins in this group [15,16]. The last type, gap junctions, represented by connexins, allow transport of small molecules and inorganic ions directly between neighbouring cells [17,18].

Therefore, from thousands of differentially expressed transcripts in porcine oocytes during in vitro maturation, we have chosen those belonging to “Biological adhesion”. In case of oocyte maturation, gap junction connections (GJCs) are of great interest, since they permit transport of compounds and substrates between an oocyte and cumulus-granulosa cells (CCs-GCs) [18]. Additionally, AJs allow sperm–oocyte fusion during fertilization [19,20]. However, the type of contact that allows post-ovulatory journey of the oocyte as well as interaction between oocyte and oviductal epithelial cells (OECs) still needs further investigation.

In this article, we presented the differences in expression of genes involved in “Biological adhesion” in porcine oocytes before and after IVM and suggested potential markers of maturational capability.

## 2. Results

Whole transcriptome profiling by Affymetrix microarray allowed us to analyse the gene expression changes in freshly isolated oocytes, before in vitro procedures (“before IVM”) in relation to after in vitro maturation (“after IVM”). By Affymetrix^®^ Porcine Gene 1.1 ST Array (Thermo Fisher Scientific, Gdansk, Poland), we have examined expression of 12,258 porcine transcripts. Genes with fold change higher than |2| and with corrected *p* values lower than 0.05 were considered as differentially expressed. This set of genes consisted of 419 different transcripts. Subsequently, the genes were used for identification of significantly enriched GO BP terms.

**Figure 1:**
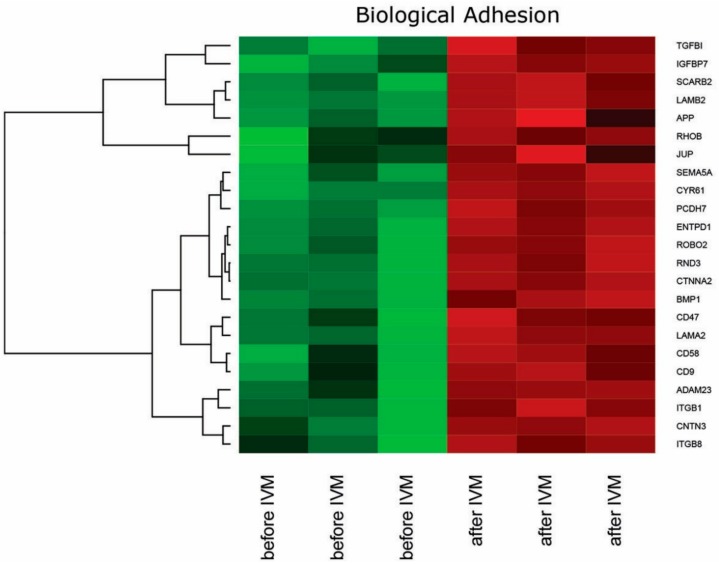
Heat map representation of differentially expressed genes belonging to the “Biological adhesion” functional category from DAVID GEOTERM BP database. Arbitrary signal intensity acquired from microarray analysis is represented by colours (green, higher; red, lower expression). Log_2_ signal intensity values for any single gene were resized to Row Z-Score scale (from −2, the lowest expression to +2, the highest expression for single gene).

DAVID (Database for Annotation, Visualization and Integrated Discovery) software was used for extraction of the genes belonging to “Biological adhesion” gene ontology Biological Process term (GO BP). We found that 23 genes from “Biological adhesion” GO BP term were significantly represented in down-regulated gene set. This set of genes was subjected to hierarchical clustering and presented as heat map (Figure 1).

Set of the differentially expressed genes belonging to “Biological adhesion” GO BP term with their official gene symbols, fold changes in expression, Entrez Gene IDs and corrected *p* values were shown (Table 1).

STRING-generated interaction network was created with differentially expressed genes belonging to the “Biological adhesion” ontology group. The intensity of the edges reflects the strength of interaction score. Applied prediction methods: text mining, co-expression, experimentally observed interactions.

**Figure 2:**
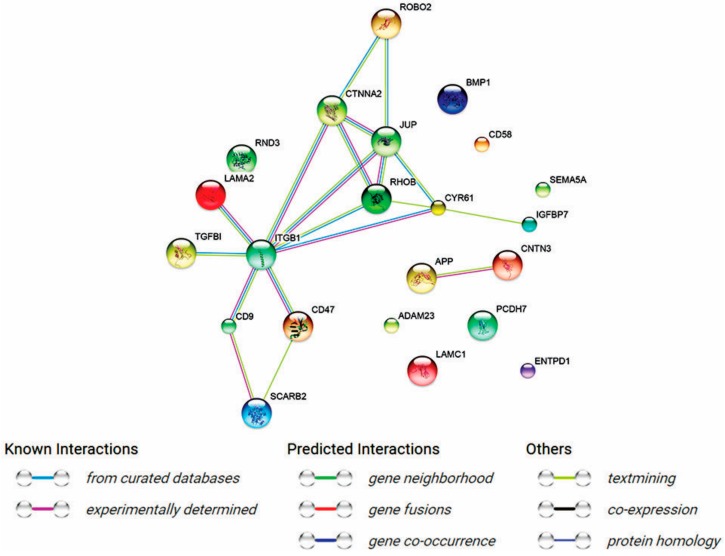
STRING-generated interaction network among differentially expressed genes belonging to the “Biological adhesion” ontology group. The intensity of the edges reflects the strength of interaction score. Applied prediction methods: text mining, co-expression, experimentally observed interactions.

Subsequently set of differentially expressed genes from “Biological adhesion” GO BP term category, were applied to STRING software version 10.5 (Search Tool for the Retrieval of Interacting Genes/Proteins, STRING Consortium 2017) for interactions prediction. Using such prediction methods provided us with molecular interaction network formed between genes of interest. In obtained results the strongest interactions were observed between *ITBG1*, *CTNNA2*, *JUP* and *RHOB* (Figure 2).

**Figure 3:**
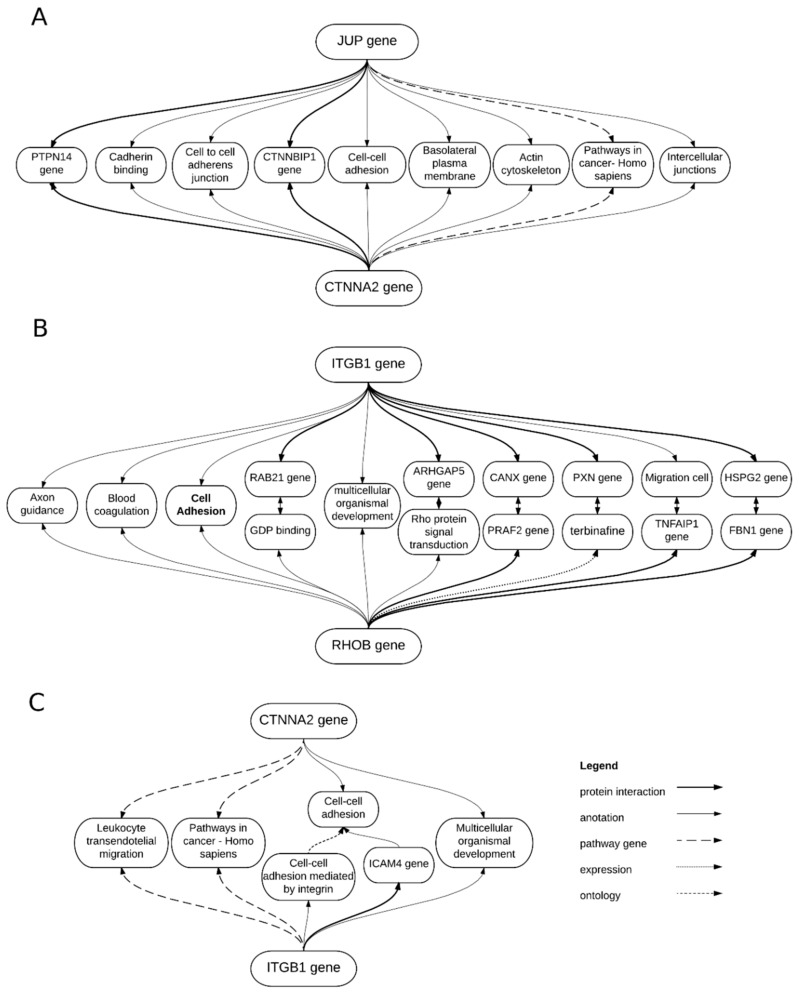
BioGraph gene interactions between (**A**) *JUP* and *CTNNA2*, (**B**) *ITGB1* and *RHOB*, and (**C**) *CTNNA2* and *ITGB1*, presenting their common processes and intermediating genes.

Specific gene interactions between those four genes were examined further using BioGraph web services. Results of such analysis were presented in Figure 3.

**Figure 4:**
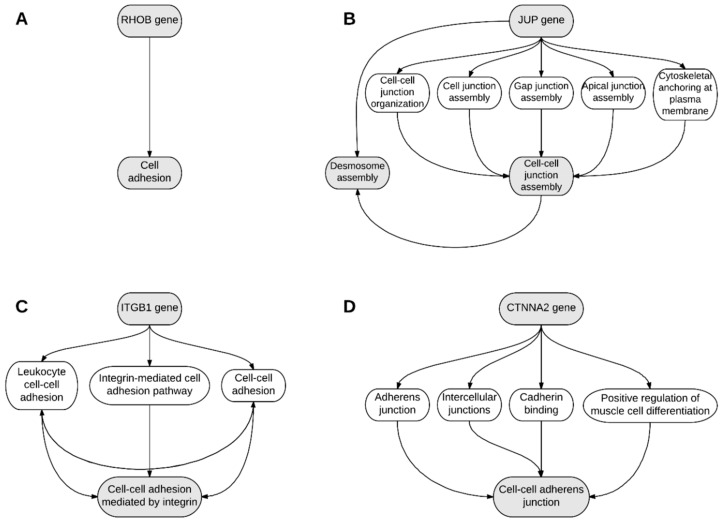
BioGraph results showing known interaction that connect the role of (**A**) *RHOB* gene in cell adhesion, (**B**) *JUP* gene in desmosome assembly, (**C**) *ITGB1* gene in cell–cell adhesion mediated by integrin, and (**D**) *CTNNA2* gene in cell to cell adherent junctions.

According to BioGraph software there are relations between *JUP* and *CTNNA2*, *ITGB1* and *RHOB* and *CTNNA2* and *ITGB1*, but not between *RHOB* and *JUP*. The adhesion-related *ITBG1*, *CTNNA2*, *JUP* and *RHOB* genes functions were further studied with BioGraph software and shown in Figure 4.

**Figure 5:**
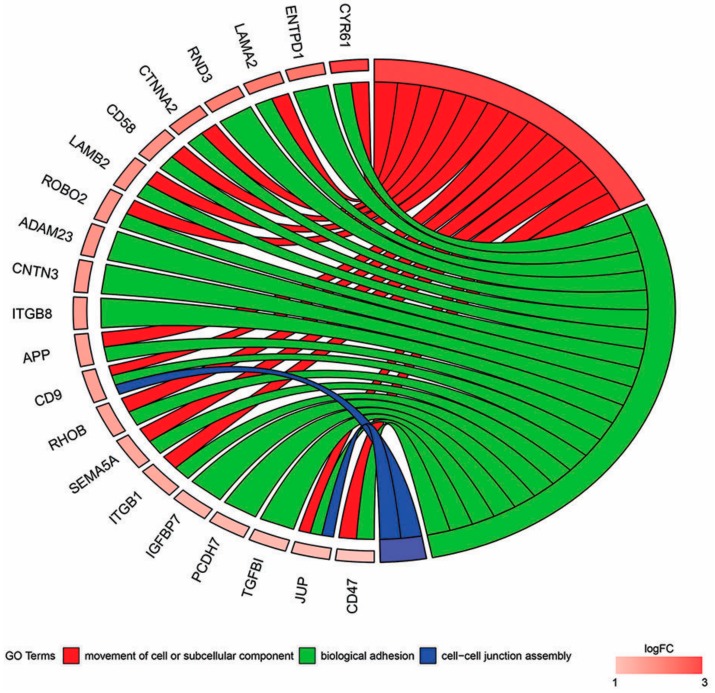
The representation of relationship between down-regulated genes that belong to the “Biological adhesion”, “Movement of cell or subcellular component”, and “Cell–cell junction assembly” GO terms. The ribbons show which genes belong to which categories. The genes were sorted by logFC from the most, to the least changed.

In Gene Ontology database genes that formed one particular GO group can also belong to other different GO term categories. For this reason, we have performed functional enrichments of GO terms based on previously uploaded gene set from “Biological adhesion” GO BP term. Down-regulated genes from “Biological adhesion” GO term belong also belong to two other GO terms from Biological Process domains. However, more GO terms from Cellular Component GO domain share the same genes with “Biological adhesion” term. Only “Movement of cell or subcellular component” and “Cell–cell junction assembly” biological process GO terms share significant amount of differently expressed genes with “Biological adhesion” GO term. The relations between these genes are shown in Figure 5.

**Figure 6:**
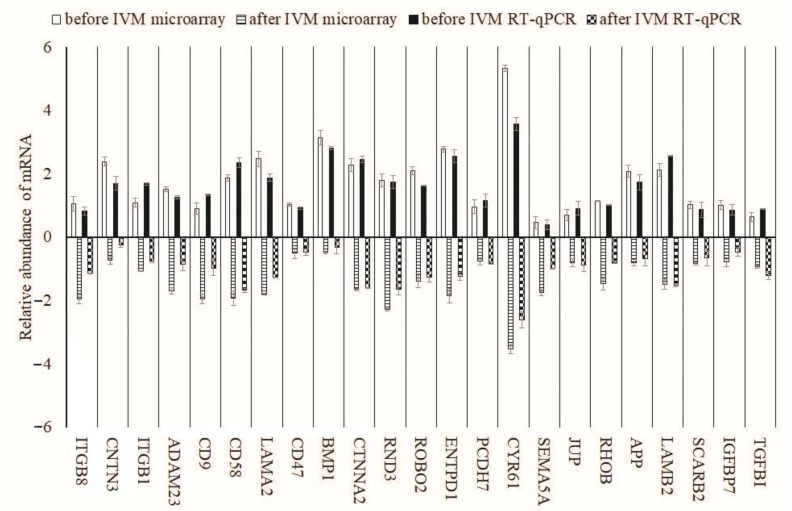
Validation of microarray data by RT-qPCR. Comparison of gene expression analysis of oocytes before in vivo and matured in vitro using microarray and RT-qPCR. RT-qPCR analysis was normalized to the expression of two housekeeping genes (*PBGD* and *ACTB*).

After RT-qPCR, we validated the gene expression profile using quantitative methods (Figure 6). We found an increased expression of *CYR61* mRNA in porcine oocytes analysed both before and after IVM, as compared to other genes (*p* < 0.001). The rest of the analysed genes displayed similar levels of expression profiles. We observed higher expression of *CD9*, *CD58*, *CTNNA2*, *PCDH7*, *JUP*, *LAMB2* and *TGFB1* after using RT-qPCR as compared to microarray quantification; however, the differences were not statistically significant. Only *CYR61* mRNA manifested increased expression after microarray analysis compared to RT-qPCR, that was statistically relevant (*p* < 0.01). When comparing these two quantification methods, we found slightly higher expression of genes when analysed using RT-qPCR compared to microarray.

## 3. Discussion

The success of in vitro fertilization (IVF) is measured by offspring outcome, however, all previous stages starting from oocytes’ selection and maturation, through fertilization, ending in blastocyst formation and embryo development are essential for final success. Among them, we focused on in vitro maturation (IVM) process, since propriety of this step is a key to successful fertilization [21], and still the outcome of completely mature and fertilizable oocytes in many mammalian species, including pigs, is insufficient [21]. During maturation, the oocyte undergoes extensive morphological and biochemical changes, including RNA accumulation required for future protein synthesis during early embryogenesis [22]. These nuclear alterations, contributing to organelles reorganization in mammalian oocytes, both in vivo and in vitro, still need further investigation. As we showed previously [8,9,10,11,23], transcriptomic profile of gene expression differs significantly in porcine oocytes before and after IVM. Thus, to improve the quantity of mature and fertilizable oocytes, as well as IVF outcome, finding new molecular markers of maturation capability is highly recommended [24,25,26,27].

This study was aimed to analyse gene expression in porcine oocytes before and after IVM in order to define differentially expressed genes, potentially involved in oocyte maturation competence. We selected genes related to the “Biological adhesion” ontology group that were significantly down-regulated after IVM as compared to before IVM.

The novelty of this study was based on brilliant cresyl blue (BCB) test application. Only BCB^+^ oocytes were selected, before and after IVM, for transcriptomic profile analyses. Because of this, we measured influence of IVM process on transcriptomic profile of oocytes of the same developmental competence. Thus, we could assume that observed changes in genes expression were triggered solely by IVM, and not by varying competence of oocytes for development.

Usually, an organism, for its development and maintenance, requires various cell–cell and cell–extracellular matrix interactions, collectively called adhesion interactions. This phenomenon can promote multiple and even opposing cellular processes, depending on cell types and circumstances. Among adherens junctions (AJs), desmosomes, tight junctions and gap junction connections (GJCs), the latter play an essential role in process of oocyte maturation and resumption of meiosis, since GJCs enable bidirectional transfer of small compounds and substrates between oocyte and somatic cumulus cells (CCs) [28,29]. During maturation, CCs undergo substantial expansion and loose tight contact with an oocyte [30]. Another essential interaction is sperm–mature oocyte fusion during fertilization, enabled by integrins, belonging to AJs [19,20].

Among all our analysed genes, the majority was related to biological adhesion processes in various systems. They were predominantly involved in organization of actin cytoskeleton and producing branching processes (*RND3* [31], *ITGB8* [32], *CYR61* [33]), migration (*TGFBI* [34], *RHOB* [35]), proliferation (*RND3* [36], *IGFBP7* [37], *CYR61* [33], *TGFBI* [38], *JUP* [39]), differentiation (*RND3* [40], *IGFBP7* [37], *CYR61* [33], *TGFBI* [38]), apoptosis (*RND3* [41], *IGFBP7* [42], *CYR61* [33]), survival (*ITGB8* [32], *CYR61* [33]), or angiogenesis (*IGFBP7* [43], *CYR61* [33]). Some were also related to mediation of bacterial adhesion and evasion (*SCARB-2* [44]), and tumorigenesis (*RHOB* [45,46,47], *IGFBP7* [48], *JUP* [39]). Despite being found in several organs, including the ovary, their role in oocyte maturation and susceptibility for fertilization has never been described before.

However, some of them have been already correlated with reproductive events. Christenson et al. (2013), using microarray assays in bovine, found significantly up-regulated expression of *RND3* (Rho Family GTPase 3) in antral, membrane-associated granulosa, and theca cells (141-, 40- and three-fold change, respectively) after comparing to before GnRH-inducted LH surge [49]. Thus, we can speculate that immature bovine oocytes are surrounded by GCs with lower *RND3* expression. This stays in accordance with previously described data, suggesting that lower *RND3* expression can enable focal adhesion [36], which is required for oocyte–GCs contact before reaching maturity. Conversely, in our study, expression of *RND3* was down-regulated after LH-supplemented IVM, however without proper estimation of total amount of that protein; it is hard to clearly explain this discrepancy.

Integrin Subunit Beta 1 (*ITGB1*) gene has been associated with embryonic processes, including implantation and trophoblastic function [50]. *ITGB1* is constitutively expressed in all stages of bovine preimplantation embryo development [51]. He et al. (2012) found higher *ITGB1* expression in biparous vs. single-bearing Mongolian sheep ovaries [52]. In the latter case, as well as in our study, describing the exact function of *ITGB1* in folliculogenesis, oocytes’ maturation and ovulation requires further investigation.

The Ras Homolog Family Member B (RHOB) is a small protein, belonging to the Rho GTPases family. It localizes in adherens junctions between oocyte and granulosa cells, with E-cadherin assigned to oocytes, and N-cadherin more to GCs [53,54]. Studies of Vega et al. (2015) on prostate cancer cell lines indicated that RhoB influences cadherin level, as they observed RhoB depletion reduced cadherins level [55]. This could explain the decreased expression of *RHOB* gene in oocytes analysed after IVM, compared to those before IVM, since contact between those cells is much looser after maturation, when COCs present their dispersed form [56].

Although integrin beta 8 (ITGB8) was not previously found to be related to oocyte–cumulus cells interactions, nor fertilization capacity, it was associated with endometrial cells’ receptivity for embryo implantation, since high expression was observed in endometrial epithelial cells [57]. The ITGB8 can trigger focal adhesion kinase (FAK), which is essential for blood vessels formation during embryonic development [58]. ITGB8, in complex with α5 integrin (α5β8), was recognized as a receptor for the extracellular matrix protein fibronectin (FN) [59]. Among many splicing variants of FN, some are also present in cumulus cells [51]. The FN was shown to negatively influence fertilization and sperm penetration in bovine COCs by interaction with spermatozoa [60]. Thus, lowered expression of *ITGB8* after IVM may reflect dispersed structure of COCs. On the other hand, it may also affect lower expression of α5β8 complex receptor, thus avoiding FN negative influence on fertilization capacity.

Insulin-like growth factor binding protein 7 (IGFBP7) is present in follicular fluid of rat follicles, supposedly suppressing estrogen production in GCs [61]. It is expressed in porcine GCs in the large antral follicles [62] and bovine corpus luteum [63]. It is supposed to act as an antagonist of activin, thus modulating development of follicles towards ovulation [61]. It was shown that IGFBP7 secreted into the corpus luteum tissue may inhibit VEGFA-stimulated angiogenesis in the luteinizing ovary after ovulation [43].

Cysteine rich angiogenic inducer 61 (CYR61) protein expression was also up-regulated in corpus luteum, associated with a switch to angiogenic phenotype. It was determined that luteal-derived endothelial cells, as well as luteal steroidogenic cells, are sources of CYR61 [64]. CYR61 was found to be up-regulated after stimulation with estrogens (17β-estradiol) [65,66], while the pre-ovulatory estrogen surge, does not stay in accordance with decreased *CYR61* level in oocytes after IVM.

Another, large group of genes was related to processes associated with cell–cell recognition and adhesion, predominately in the nervous system, such as axonal growth, myelination, neurotransmission, formation and maintenance of neuron type-specific networks in the brain. The group included: *APP* (amyloid beta precursor protein) [67], *ENTPD1* (Ectonucleoside triphosphate diphosphohydrolase 1) [68], *PCDH7* (Protocadherin 7) [69], *SEMA5A* (Semaphorin A) [70], *CNTN3* (Contactin3) [71], *ROBO2* (Roundabout guidance receptor 2) [72], *CTNNA2* (Catenin alpha 2) [73], and *ADAM23* (A member of disintegrin and metalloprotease domain family) [74,75]. The only gene indirectly linked to oocyte maturation was *APP*, responsible for amyloid plaques formation in the brain of patients with Alzheimer’s disease [67]. Khan et al. (2016) hypothesized APP could be a potential biomarker of follicle differentiation and predictor of oocyte competence in cows, since they found association between diverse FSH doses and APP expression levels in oocytes [76].

There were also two genes encoding proteins with roles associated mainly with the immune system. The first one was lymphocyte function-associated antigen 3 (CD58, LFA-3), which is the ligand for T lymphocyte CD2 protein, thus facilitating T lymphocyte adhesiveness [77]. CD58 expression was immunohistochemically recognized on human granulosa cells in secondary growing follicles and preovulatory follicles, with expression increasing after ovulation in large luteal cells in the midluteal phase [78]. However, there is no data concerning CD58 expression in oocytes, and we cannot correlate the mentioned results with GCs–oocyte adhesion, since we found *CD58* expression down-regulated in oocytes after IVM. The second one, integrin-associated protein (CD47, IAP), increases intracellular calcium concentration upon cell adhesion to extracellular matrix and is involved in immune regulation, by preventing maturation of dendritic cells (DCs) and blocking cytokine production by matured DCs [79]. Moreover, CD47 is a receptor for the thrombospondin (THBS) family of proteins [80], which act as inhibitors of angiogenesis (anti-VEGF action) [81]. Previous studies showed increasing expression of CD47 in human granulosa and theca interna cells during final follicle maturation, corpus luteum (CL) formation and luteal function, suggesting that CD47 plays role in angiogenesis regulation, necessary for the formation and function of the follicle and CL [82].

Decreased expression was also determined for *CD9* (member of the transmembrane-4 tetraspanin superfamily), *BMP1* (Bone Morphogenetic Protein 1), *LAMA2* (laminin subunit α-2) and *LAMB2* (laminin β-2) genes belonging to “Biological adhesion” ontology group. This set of genes has also been previously identified in “Bone development”, “Cellular components of morphogenesis”, and “Cell migration” ontology groups. Among them, *CD9* and *BMP1* have the most relevant function to events in the reproductive tract, participating in oocyte–sperm fusion, and taking part in oocyte nuclear and cytoplasmic maturation, respectively. Wider descriptions of possible implications in oocyte maturation have already been done in our previous reports [8,9,11].

## 4. Materials and Methods

### 4.1. Experimental Design

Oocytes were collected and subjected to two Brilliant Cresyl Blue (BCB) tests and divided into two groups. The first group (“before IVM”) included oocytes graded as BCB-positive (BCB^+^) and directly exposed to microarray assay and RT-qPCR. The second group (“after IVM”) included BCB^+^ oocytes which were then matured in vitro, and if classified as BCB^+^ in second BCB test passed to molecular analyses.

### 4.2. Animals

A total of 45 pubertal crossbred Landrace gilts bred on a commercial local farm were used in this study. They had a mean age of 155 days (range 140–170 days) and a mean weight of 100 kg (95–120 kg). All animals were bred under the same conditions and fed the same forage (depending on age and reproductive status). All experiments were approved by the Local Ethic Committee on 1 June 2012 with resolution number 32/2012.

### 4.3. Collection of Porcine Ovaries and Cumulus–Oocyte Complexes (COCs)

The ovaries and reproductive tracts were recovered at slaughter and transported to the laboratory within 40 min. at 38 °C in 0.9% NaCl. To provide optimal conditions for subsequent oocyte maturation and fertilization in vitro, the ovaries of each animal were placed in a 5% fetal bovine serum solution (FBS; Sigma-Aldrich Co., St. Louis, MO, USA) in PBS. Single large follicles (>5 mm) were opened by puncturing with a 5 mL syringe and 20-G needle in a sterile Petri dish, and COCs were recovered. The COCs were washed three times in modified PBS supplemented with 36 µg/mL pyruvate, 50 µg/mL gentamicin, and 0.5 mg/mL BSA (Sigma-Aldrich, St. Louis, MO, USA). The COCs were selected under an inverted microscope Zeiss, Axiovert 35 (Lübeck, Germany), counted, and morphologically evaluated. Only COCs of grade I, possessing homogeneous ooplasm and uniform, compact cumulus cells, were considered for further use, resulting in a total of 300 grade I oocytes (3 × *n* = 50 “before IVM” group, 3 × *n* = 50 “after IVM” group).

### 4.4. Assessment of Oocyte Developmental Competence by BCB Test

Brilliant Cresyl Blue (BCB) test was used for assessment of porcine oocytes’ quality and maturity [83]. The glucose-6-phosphate (G6PDH) enzyme converts BCB stain from blue to colourless. In oocytes that completed the growth, activity of the enzyme decreases and the stain cannot be reduced, resulting in blue oocytes (BCB^+^). To perform the BCB staining test, oocytes were washed twice in modified Dulbecco’s Phosphate Buffered Saline (DPBS), commercially supplemented with 0.9 mM calcium, 0.49 mM magnesium, 0.33 mM pyruvate, and 5.5 mM glucose (Sigma-Aldrich, St. Louis, MO, USA), and additionally with 50 IU/mL penicillin, 50 µg/mL streptomycin (Sigma-Aldrich, St. Louis, MO, USA), and 0.4% bovine serum albumin (BSA) [*w*/*v*] (Sigma-Aldrich, St. Louis, MO, USA). They were then treated with 13 µM BCB (Sigma-Aldrich, St. Louis, MO, USA) diluted in DPBS at 38.5 °C, 5% CO_2_ for 90 min. After treatment, the oocytes were transferred to DPBS and washed twice. During washing, the oocytes were examined under an inverted microscope and classified as stained blue (BCB^+^), or colourless (BCB^−^). Only the granulosa cell-free BCB^+^ oocytes were used for subsequent molecular analyses (“before IVM” group), or IVM, followed by second BCB test and molecular analyses (“after IVM” group).

### 4.5. In Vitro Maturation of Porcine Cumulus-Oocyte-Complexes (COCs)

After the first BCB test, the BCB^+^ COCs were subjected to IVM. The COCs were cultured in Nunclon™Δ 4-well dishes (Thermo Fisher Scientific, Waltham, MA, USA) in 500 μL standard porcine IVM culture medium: TCM-199 (tissue culture medium) with Earle’s salts and l-glutamine (Gibco BRL Life Technologies, Grand Island, NY, USA), supplemented with 2.2 mg/mL sodium bicarbonate (Nacalai Tesque, Inc., Kyoto, Japan), 0.1 mg/mL sodium pyruvate (Sigma-Aldrich, St. Louis, MO, USA), 10 mg/mL BSA (Bovine Serum Albumin) (Sigma-Aldrich, St. Louis, MO, USA), 0.1 mg/mL cysteine (Sigma-Aldrich, St. Louis, MO, USA), 10% (*v*/*v*) filtered porcine follicular fluid, and gonadotropin supplements at final concentrations of 2.5 IU/mL hCG (human Chorionic Gonadotropin) (Ayerst Laboratories, Inc., Philadelphia, PA, USA) and 2.5 IU/mL eCG (equine Chorionic Gonadotropin) (Intervet, Whitby, ON, Canada). Wells were covered with mineral oil overlay and cultured at 38 °C under 5% CO_2_ in air for 22 h, and then for additional 22 h in medium without hormones. After cultivation, the second BCB staining test was performed, and BCB^+^ oocytes were used for further molecular analyses.

### 4.6. RNA Extraction from Porcine Oocytes

Total RNA was extracted from samples using TRI Reagent (Sigma, St Louis, MO, USA) and RNeasy MinElute cleanup Kit (Qiagen, Hilden, Germany). The amount of total mRNA was determined using optical density at 260 nm, and the RNA purity was estimated using the 260/280 nm absorption ratio (higher than 1.8) (NanoDrop spectrophotometer, Thermo Scientific, ALAB, Poland). The RNA integrity and quality were checked on a Bioanalyzer 2100 (Agilent Technologies, Inc., Santa Clara, CA, USA). The resulting RNA integrity numbers (RINs) were between 8.5 and 10 with an average of 9.2. The RNA in each sample was diluted to a concentration of 100 ng/μL with an OD260/OD280 ratio of 1.8/2.0. From each RNA sample, 500 ng of RNA were taken. The remaining amount of isolated RNA was used for RT-qPCR study.

### 4.7. Microarray Expression Analysis and Statistics

Experiments were performed in three replicates. Total RNA (100 ng) from each pooled sample was subjected to two round sense cDNA amplification (Ambion^®^ WT Expression Kit, Thermo Fisher Scientific, Gdansk, Poland). The obtained cDNA was used for biotin labeling and fragmentation by Affymetrix GeneChip^®^ WT Terminal Labeling and Hybridization (Affymetrix, Thermo Fisher Scientific, Gdansk, Poland). Biotin-labeled fragments of cDNA (5.5 μg) were hybridized to Affymetrix^®^ Porcine Gene 1.1 ST Array Strip (48 °C/20 h). Then, microarrays were washed and stained according to the technical protocol, using Affymetrix GeneAtlas Fluidics Station. The array strips were scanned, employing Imaging Station of GeneAtlas System. The preliminary analysis of the scanned chips was performed, using Affymetrix GeneAtlas^TM^ Operating Software. Quality of gene expression data was checked according to quality control criteria provided by the software. Obtained CEL files were imported into downstream data analysis software.

All analyses were performed using BioConductor software (Open Source Software For Bioinformatics), based on the statistical R programming language. For background correction, normalization and summation of raw data, the Robust Multiarray Averaging (RMA) algorithm, implemented in “affy” package of BioConductor, was applied. Biological annotation was taken from BioConductor “oligo” package, where annotated data frame object was merged with normalized data set, resulting in a complete gene data table. Statistical significance of analysed genes was performed by moderated t-statistics from the empirical Bayes method. Obtained *p* value was corrected, for multiple comparisons, using the Benjamini and Hochberg’s false discovery rate. The selection of significantly changed gene expression was based on *p* value beneath 0.05 and expression fold higher than |2|.

Functional annotation clustering of differentially expressed genes was performed using DAVID (Database for Annotation, Visualization and Integrated Discovery). Gene symbols for up- or down-regulated genes, from each of the compared groups were loaded to DAVID by “RDAVIDWebService” BioConductor package. In this analysis, we focused on one GO term group described as “Biological adhesion” that was separated from other GO groups and subjected to hierarchical clustering algorithm, and presented as a heat map graph. “Biological adhesion” GO term (GO:0022610) belongs to Biological Process GO domain. “Biological adhesion” is defined as “The attachment of a cell or organism to a substrate, another cell, or other organism. Biological adhesion includes intracellular attachment between membrane regions”. It contains 29,306 annotated genes, of which 1849 are annotated to *Sus scrofa domestica*.

Interactions between differentially expressed genes/proteins belonging to “Biological adhesion” ontology group were investigated by STRING10 software (Search Tool for the Retrieval of Interacting Genes). List of gene names were used as query for interaction prediction. Searching criteria were based on co-occurrences of genes/proteins in scientific texts (text mining), co-expression and experimentally observed interactions. The results of such analysis generated gene/protein interaction network where the intensity of the edges reflects the strength of interaction score. Besides interaction prediction, STRING also allowed us to perform functional enrichments of GO terms based on previously uploaded gene set from “Biological adhesion” GO BP term.

Genes with the strongest interactions from STRING analysis were subjected to BioGraph web services. BioGraph is based on the integration of biomedical knowledge bases and yields intelligible and literature-supported indirect functional relations.

### 4.8. Validation of Microarray Results with Real-Time Quantitative Polymerase Chain Reaction (RT-qPCR)

RT-qPCR analysis was performed in order to validate microarray results, using the same RNA samples as for microarray profiling experiments. Tests were performed in three replicates.

Total RNA was isolated from oocytes before and/or after IVM. The RNA samples were re-suspended in 20 µL of RNase-free water and stored in liquid nitrogen. Afterwards, they were treated with DNase I and reverse-transcribed (RT) into cDNA. RT-qPCR was conducted in a Light Cycler real-time PCR detection system (Roche Diagnostics GmbH, Mannheim, Germany) using SYBR^®^ Green I as a detection dye, and target cDNA was quantified using the relative quantification method. For amplification, 2 µL of cDNA solution was added to 18 µL of QuantiTect^®^ SYBR^®^ Green PCR (Master Mix Qiagen GmbH, Hilden, Germany) and primers (Table 2). One RNA sample of each preparation was processed without the RT-reaction to provide a negative control for subsequent PCR. The relative abundance of *TGFBI*, *IGFBP7*, *SCARB2*, *LAMB2*, *APP*, *RHOB*, *JUP*, *SEMA5A*, *CYR61*, *PCDH7*, *ENTPD1*, *ROBO2*, *RND3*, *CTNNA2*, *BMP1*, *CD47*, *LAMA2*, *CD58*, *CD9*, *ADAM23*, *ITGB1*, *CNTN3*, and *ITGB8* transcripts in each sample was standardized to the glyceraldehyde-3-phosphate dehydrogenase (*PBGD*) and actin beta (*ACTB*) internal standards.

## 5. Conclusions

All analysed genes presented decreased mRNA expression after IVM, compared to before IVM. However, the extent and how the mentioned genes affect oocyte morphology during the complex process of maturation are not yet known. Although only few of them were associated with reproductive events, including oocyte maturation and fertilization feasibility, they may be potential markers of oocyte developmental competence in pigs. To confirm their involvement in oogenesis and other regulatory processes such as fertilization and embryogenesis, further protein assays are required.

## Figures and Tables

**Table 1:** Official genes symbols, fold changes, adjusted *p* values and ENTREZ Gene ID of differentially expressed genes belonging to the “Biological adhesion” functional category from DAVID GEOTERM BP database.

Name	Fold	adj.*p*.Value	ENTREZ Gene ID
ITGB8	3.240231	1.198584 × 10^−2^	3696
CNTN3	3.369908	3.018953 × 10^−3^	5067
ITGB1	2.730502	3.705215 × 10^−3^	3688
ADAM23	3.519362	7.243130 × 10^−3^	8745
CD9	3.036900	6.332387 × 10^−3^	928
CD58	3.625447	4.641977 × 10^−3^	965
LAMA2	4.552369	7.946265 × 10^−4^	3908
CD47	2.054594	9.288999 × 10^−3^	961
BMP1	3.586853	5.391380 × 10^−4^	649
CTNNA2	4.349240	5.121808 × 10^−4^	1496
RND3	4.413617	6.618426 × 10^−4^	390
ROBO2	3.524243	1.183495 × 10^−3^	6092
ENTPD1	4.796997	3.276430 × 10^−4^	953
PCDH7	2.382089	6.329369 × 10^−4^	5099
CYR61	12.398174	7.535476 × 10^−5^	3491
SEMA5A	2.829721	1.092396 × 10^−3^	9037
JUP	2.264561	3.461543 × 10^−2^	3728
RHOB	2.959342	2.898885 × 10^−2^	388
APP	3.085100	5.602323 × 10^−3^	351
LAMB2	3.586055	1.879115 × 10^−4^	3913
SCARB2	2.477578	1.415004 × 10^−3^	950
IGFBP7	2.476722	2.496043 × 10^−3^	3490
TGFBI	2.282538	1.912689 × 10^−3^	7045

**Table 2:** Oligonucleotide sequences used for RT-qPCR analysis.

Name	Gene Accession Numer	Primer Sequence (5′-3′)	Product Size (bp)
ITGB8	NM_002214.2	AAGGGCCAAGTGTGTAGTGG TCTGACATTTGGTCCGCATA	233
CNTN3	XM_021069229	GAATGTTTTGCCCTTGGAAA GCAGCCCATCACTTCTTCTC	61
ITGB1	NM_214015.2	ACCATGCCAATTTCTGCCTGG AACGCACGATCATGTTGGA	208
ADAM23	XM_021076147	GAATCACAGCATGGAAAGCAGT GCATGAGAAGAGCGACAC	179
CD9	NM_214006.1	CAAAGGGACGTACTCTCAAGC GACCCCGAGAAGATGACCAA	249
CD58	NM_213795	CAGTACTGCCAGCGGTGATAT GGAGGCATCGGTAATAAGG	202
LAMA2	XM_013992573.1	GATACAAATGACCCCGTGTTAA TCGAATACAACCTCGGAA	95
CD47	NM_213982.1	TGGAGCCATTCTTTTCATCC AATCAGAAGAGGGCCATGC	241
BMP1	XM_021072336.1	AGCTCTTCGACGGTTACGACA ACAGAATCTCCCGCCGAGT	93
CTNNA2	XM_013995995.1	CCTACCTTCAACGGATTGCCCTT CTGATACTTTGTTGAGGC	204
RND3	NM_214296.1	CCCAACACCAAAATGCTCTTAA GTGGCTGCTCCAATCTGT	145
ROBO2	XM_013982523.1	GGAACAGCTTCTTCTAAGGGAA TAAAGAAATTGTTCATTGCACT	238
ENTPD1	NM_214153.1	GCTATGGGAAGGATCAAGCAGT GCAGGGAGCCTCATAAAG	139
PCDH7	NM_001244484.1	TTCCACTCCCAGAGGACAACG GTCAGGGCTACATCTGGAA	83
CYR61	XM_001927740.4	CCAATGACAACCCCGACTGCCC GGTACTTCTTCACGCTGG	176
SEMA5A	XM_013984924.1	AACACCAGCATAACCAACCACA ACTGGGGAATTACAAGAAGC	221
JUP	NM_214323.1	ATCCCATGGACACCTACAGCGG CTCAGGCACTTTGCTATC	148
RHOB	NM_001123189.1	TATGTGCTTCTCGGTGGACACG AGGTAGTCGTAGGCTTGG	230
APP	NM_214372	TGGGGAAAGACACAAACCCTT CATGCACTAGTTTGATACAGCTT	206
LAMB2	XM_013981664.1	GCTGCCCAAGGATGACCACAT CCTCCTGTTCGCACTAGCTT	130
SCARB2	NM_001244155.1	AGTCGCCTGAAGTCTGTGGT AGTTGCCCCATGTCGTAGTC	236
IGFBP7	NM_001163801.1	ATAACCTGGCCATTCAGACG ACAGCTCAGCACCTTCACCT	207
TGFBI	NM_214015.2	ACCATGCCAATTTCTGCCTGGA ACGCACGATCATGTTGGA	208
PBGD	NM_001097412.1	GAGAGTGCCCCTATGATGCTAT GATGGCACTGAACTCCT	214
ACTB	XM_003124280.3	CCCTTGCCGCTCCGCCTTCGCA GCAATATCGGTCATCCAT	69
